# Editorial: Potential effects and mechanisms of bone homeostasis on tumor bone metastasis

**DOI:** 10.3389/fendo.2023.1256406

**Published:** 2023-07-26

**Authors:** Jiyu Han, Zitong Zhao, Daqian Wan

**Affiliations:** ^1^ Department of Orthopedics, Tongji Hospital, School of Medicine, Tongji University, Shanghai, China; ^2^ Key Laboratory of Spine and Spinal Cord Injury Repair and Regeneration, Ministry of Education, Shanghai, China; ^3^ Department of Anesthesiology and Pain Rehabilitation, Shanghai YangZhi Rehabilitation Hospital (Shanghai Sunshine Rehabilitation Center), School of Medicine, Tongji University, Shanghai, China

**Keywords:** bone homeostasis, bone metastasis (BM), cancer, tumor, endocrinology

The bone is important for supporting body movement. However, the number of bone injuries caused by tumor disease, trauma, and osteoporosis is increasing dramatically every year. All of these orthopedic diseases can have serious health consequences, and bone metastasis is the most predominant complication of malignant tumors. In tumor bone metastasis, the bone represents “an airport hub” of cancer cells derived from different types of primary tumors. Interactions taking place in this microenvironment can determine cell fate that impacts on the clinical outcomes of the cancer. Alterations to bone homeostasis can particularly favor tumor homing and consequent osteolytic or osteoblastic lesions. When tumor cells are established in the bone microenvironment by disrupting bone homeostasis, it will result in increased bone destruction (osteoclast) and/or bone formation (osteoblast). Overall, tumor cells interact with bone and bone marrow cells, disrupting bone homeostasis and driving tumor growth. This process of bone remodeling increases the risk of fractures and causes severe bone pain. Since then, studying bone homeostasis and how it relates to tumor bone metastasis will allow researchers to identify future directions in orthopedic disease research.

One potential effect of altered bone homeostasis on tumor bone metastasis is the creation of a conducive “premetastatic niche.” Tumors release factors that prime the bone microenvironment, preparing it for the arrival and survival of circulating tumor cells. This process involves the recruitment and activation of bone marrow-derived cells, the remodeling of the extracellular matrix, and the release of growth factors. Disruptions in the delicate balance of bone remodeling could enhance these premetastatic changes, facilitating tumor cell colonization.

Furthermore, bone homeostasis plays a critical role in regulating immune responses within the bone microenvironment. Osteoblasts and osteoclasts contribute to immune cell recruitment and activation, influencing immune surveillance against tumor cells. Dysregulation of bone remodeling processes may impair immune cell function, leading to immune evasion and promoting the survival and growth of tumor cells in the bone microenvironment.

Additionally, bone-derived factors and signaling pathways have been found to contribute to tumor growth and progression. Cytokines, chemokines, and growth factors produced by bone cells can directly or indirectly influence tumor cell behavior, promoting their survival and proliferation within the bone microenvironment. Imbalances in bone homeostasis may result in aberrant signaling, favoring tumor growth and metastasis.

Understanding the potential effects and mechanisms of bone homeostasis on tumor bone metastasis provides a foundation for the development of targeted therapeutic interventions. By targeting the bone microenvironment and associated signaling pathways, we may disrupt the vicious cycle between tumor cells and the bone, potentially preventing or limiting the spread of cancer. Promising strategies, such as bisphosphonates, RANKL inhibitors, and immune-based therapies, are being explored in preclinical and clinical studies to modulate bone homeostasis and improve outcomes for patients with bone metastasis.

A total of five articles were included in this Research Topic, including three original research articles and two review articles.

Zhao et al. introduced differentially expressed genes associated with breast cancer bone metastasis. These differentially expressed genes provide further research directions for the pathogenesis and prognosis of breast cancer bone metastasis. Among them, the core genes SERPING1 and GIMAP4 may play a crucial role in bone metastasis by influencing the imbalance of bone homeostasis in the bone microenvironment. These findings shed light on the underlying mechanisms of breast cancer bone metastasis and open avenues for potential therapeutic interventions targeting the bone microenvironment.

Gao et al. developed and validated a novel nomogram that demonstrates superior predictive ability for the diagnosis and prognosis of rare male breast cancer with bone metastases (MBCBM) compared to the TNM staging system. The results of this development can serve as a valuable tool to assist doctors and patients in conducting rapid personalized risk assessments, making informed clinical decisions, and designing optimal treatment plans and follow-up strategies. This innovative approach provides a valuable resource for improving the management and outcomes of MBCBM patients.

Li et al. found that overall adjustment acupuncture (OA) improved osteoporosis and exerted a regulatory effect on the systemic estrogen levels and hypothalamic-pituitary-adrenal (HPA) axis activity in ovariectomized rats. Further studies are still needed to determine how OA regulates bone metabolism.

Zhang et al. focused on the multifunctional roles of the RANKL/RANK/OPG system. The idea is that RANKL is a potentially significant target for bone metastases treatment. Suppressing the RANKL/RANK/OPG system could present new research directions for the treatment of skeletal diseases and bone metastasis in tumors.

Martiniakova et al. reviewed the relationship between bone-derived factors and tumor bone metastasis, emphasizing their importance as prognostic biomarkers and potential therapeutic targets in bone metastasis. This review provides a foundation for a better understanding of tumor-specific pathways associated with bone metastasis and the identification of potential tumor-specific treatment targets.

The purpose of this Research Topic is to provide a platform for researchers who work on orthopaedics, cancer, and metastasis to investigate the pathological mechanism of tumor bone metastasis caused by the interruption of the homeostasis of the bone microenvironment by tumor cells. We wish to elucidate the potential roles and regulatory mechanisms of the complex crosstalk between tumor cells and the bone microenvironment. Further, we hope this Research Topic can open our minds to exploring underlying mechanisms of bone homeostasis in other orthopedic diseases.

## Author contributions

JH: Wrote the draft. JH, ZZ, and DW: Reviewed and edited the article. All authors contributed to the article and approved the submitted version.

